# Sky-canopy border length, exposure and thresholding influence accuracy of hemispherical photography for complex plant canopies

**DOI:** 10.1186/s40529-018-0235-9

**Published:** 2018-07-28

**Authors:** Guo-Zhang M. Song, Kuo-Jung Chao, David Doley, David Yates

**Affiliations:** 1 Department of Soil and Water Conservation, National Chung Hsing University, Taichung, 40227 Taiwan; 2 International Master Program of Agriculture, National Chung Hsing University, Taichung, 40227 Taiwan; 3 Centre for Mined Land Rehabilitation, The University of Queensland, Brisbane, QLD 4072 Australia

**Keywords:** Canopy openness, Digital number, Gap fraction, Leaf area index, Mixed sky-canopy pixel

## Abstract

**Background:**

Hemispherical photography (HP) is a popular method to estimate canopy structure and understorey light environment, which analyses photographs acquired with wide view-angle lens (i.e. fisheye lens). To increase HP accuracy, the approaches of most previous studies were to increase the preciseness of exposure and thresholding of photographs, while ours quantified effects of canopy properties (gap fraction and length of sky-canopy border (SCB)) and errors of exposure and thresholding on the accuracy of HP.

**Results:**

Through analysing photographs of real and model canopies, it was showed that HP inaccuracy resulted from the mismatch between exposure and thresholding rather than exposure or thresholding errors alone. HP inaccuracy was a function of the SCB length and the extent of exposure and thresholding errors, but independent of gap fraction.

**Discussion:**

In photographs, SCBs are recorded as grey pixels which greyness is in between that of sky and canopy pixels. When there are exposure and thresholding errors, grey pixels are those prone to be misclassified in image analysis. Longer (vegetation with taller canopies) and wider (lower image sharpness) SCBs in photographs can both result in a higher amount of grey pixels and ultimately higher HP inaccuracy for a given extent of exposure and threshold errors.

**Conclusions:**

Using lenses with view angle narrower rather than that of fisheye lens can shorten the SCB length in photographs and in turn reduce HP estimation inaccuracy for canopy structure and understorey light environment. Since short SCBs and low levels of exposure and thresholding errors can both result in low HP inaccuracy, to identify the true performance of new exposure and thresholding methods for HP, photographs recording canopies with long SCBs and acquired with fisheye lenses should be used. Because HP inaccuracy in a function of the amount of grey pixels resulting from SCBs, the amount of these pixels in photographs can be used as a universal parameter to quantify canopy properties influential to HP estimation and in turn make cross-study comparisons feasible.

**Electronic supplementary material:**

The online version of this article (10.1186/s40529-018-0235-9) contains supplementary material, which is available to authorized users.

## Background

Using hemispherical photography (HP) to estimate canopy parameter (e.g. canopy gap fraction and leaf area index (LAI)) relies on correct exposure and thresholding of canopy hemispherical photographs (CHPs) (Beckschäfer et al. 2013; Jonckheere et al. 2005; Rich 1990). If CHPs are overexposed or analysed with threshold values that are too low, canopy gap fraction will be overestimated and LAI will be underestimated (Zhang et al. 2005). Conversely, the underexposure of CHPs or application of too-high threshold values will result in underestimated gap fraction and overestimated LAI. Achieving correct exposure and thresholding are widely-recognised challenges in HP (Beckschäfer et al. 2013; Jonckheere et al. 2005; Rich 1990). Most previous studies attempted to improve HP accuracy by increasing the preciseness of exposure and thresholding (Rich 1990; Zhang et al. 2005). Identifying mechanisms of how exposure and thresholding errors influence HP accuracy can provide hints for novel approaches to raise HP accuracy.

There are multiple sources of grey pixels in CHPs. In CHPs, the desired sky and canopy pixels should appear white and black respectively. Nevertheless, some sky pixels look grey because of thick cloud cover and low sky illumination created by the direction of sun radiation (Rich 1990). On the other hand, pixels recording bright leaves and light-coloured trunks appear grey. The other main source of grey pixels is the so-called mixed sky-canopy pixels (hereafter mixed pixels) (Leblanc et al. 2005), which record portions of both sky and canopy in single pixels. Using regular grid cells to record digital images, widthless borders between two photographing objects (i.e. sky-canopy borders, SCBs, in the present study) are inevitably recorded as zones in which every single pixel contains images of these two objects (Fisher 1997). In the present study, these zones recording images of SCBs are defined as mixed-pixel zones (MPZs). Mixed pixels also include pixels recording images of linear (e.g. twigs) and small (e.g. small canopy openings) sub-pixel objects (Fisher 1997). Due to the nature of the grid pixel system of digital images, even with optically-perfect lens, the widthless SCBs are captured as MPZs which as wide as at least one pixel. Lens imperfections, image editing and operation errors (e.g. focusing failure, smudged lens, canopies moved by wind or camera shake) can blur SCBs and further increase the width of SCBs images.

Effects of exposure and thresholding errors on HP accuracy are exerted through their interactions with grey pixels in CHPs. Thresholding is a step of image analysis for HP, in which image pixels originally with a continuous greyness gradient are classified into either black or white pixels (binary images) by using a cut-off greyness (e.g. Rich 1990). Due to distinct differences between the greyness of sky and canopy pixels, HP inaccuracy introduced by (manual or automatic) thresholding generally results from the misclassification of grey pixels rather than sky or canopy pixels. Therefore, the extent of HP inaccuracy resulting from thresholding errors should be a function of the grey-pixel fraction. Optimum exposure allows the image capture devices to capture the desired amount of light through manipulating shutter speed, lens aperture and light sensitivity (i.e. ISO value) of the device (Allen and Triantaphillidou 2011). In HP, blooming is an over-exposure effect in which excessive light causes light saturation on the sky pixels and pixels nearby, and in turn canopy gaps appear larger than they should be (Leblanc et al. 2005). In other words, overexposure can result in that all grey pixels located between sky and canopy pixels (i.e. mixed pixels) appear as bright as sky pixels. Consequently, the extent of HP inaccuracy caused by exposure errors should also be a function of the grey-pixel fraction. Even though the misclassification of grey pixels has been widely recognised as a source of HP inaccuracy and various studies have developed new methods to classify them precisely (Hwang et al. 2016; Leblanc et al. 2005; Macfarlane 2011; Macfarlane et al. 2014), few studies quantified their effects.

The effects of exposure and thresholding errors on HP estimation have to be examined together. The digital number (also known as the greyness scale number) of all pixels in CHPs increases with exposure (Zhang et al. 2005). Nevertheless, as long as exposure is not high enough to cause the so-called blooming effect (Leblanc et al. 2005), the effects of higher exposure on HP estimates can be cancelled out by using higher threshold values in CHP analysis (Song et al. 2014). In other words, an estimation inaccuracy cannot be introduced to HP by either exposure or thresholding errors alone, but by the mismatch between exposure and thresholding (hereafter, exposure-thresholding mismatch, ETM).

HP inaccuracy is likely a function of the ETM extent and two canopy properties (gap fraction and SCB length). Macfarlane et al. (2000) and Macfarlane (2011) showed that, in Eucalyptus forests, the estimated LAI changed by 9–13% for each stop deviation from the correct exposure, suggesting that HP inaccuracy is a linear function of the ETM extent. Nevertheless, further studies are needed to show whether this relationship is applicable to other forest types. Macfarlane (2011) had reported a negative linear relationship between the grey-pixel fraction and canopy gap fraction. As mentioned above, pixels recording SCBs appear grey. Therefore, canopy gap fraction and SCB length may influence HP estimation through their effects on the grey-pixel fraction.

To identify whether canopy properties influence HP inaccuracy through their effects on the grey-pixel fraction, a model canopy system was used to minimise the sources of grey pixels and manipulate two canopy properties, SCB length and gap fraction, independently. Model canopies have been used in several HP studies because they are more controllable and manipulable than real canopies (Chianucci 2016; Hwang et al. 2016; Macfarlane et al. 2014; Song et al. 2014). In the present study, model canopies were made with opaque black adhesive vinyl sheet, which diminished the amount of grey pixels caused by bright canopy elements; the greyness variation of sky pixels was controlled by using an artificial light source. The image size of smallest canopy elements and openings of model canopies were larger than the pixel size of the camera we used, which eliminated mixed pixels resulting from sub-pixel objects. Consequently, in our model CHPs, the only source of grey pixels is mixed pixels recording SCBs. This system also allowed us to vary SCB length and gap fraction independently, so that we could distinguish one’s effects from the other’s.

The present study aimed to improve HP accuracy through quantifying the relationships between canopy properties (SCB length and gap fraction), the grey-pixel fraction, the ETM extent and HP inaccuracy. We asked the following questions: (1) Is the grey-pixel fraction in a CHP a function of canopy gap fraction and SCB length? (2) Is the linear relationship between HP inaccuracy and the ETM extent (reported by Macfarlane (2011)) applicable to all forest types? (3) Can these two canopy properties influence HP accuracy through their effects on the grey-pixel fraction?

## Materials and methods

The grid model CHPs and real CHPs used in the present study were those in Song et al. (2014). For photographs showing the design of grid model canopies and image acquisition devices of model CHPs, please refer to Song et al. (2014).

### Design of model canopy

**Fig. 1:**
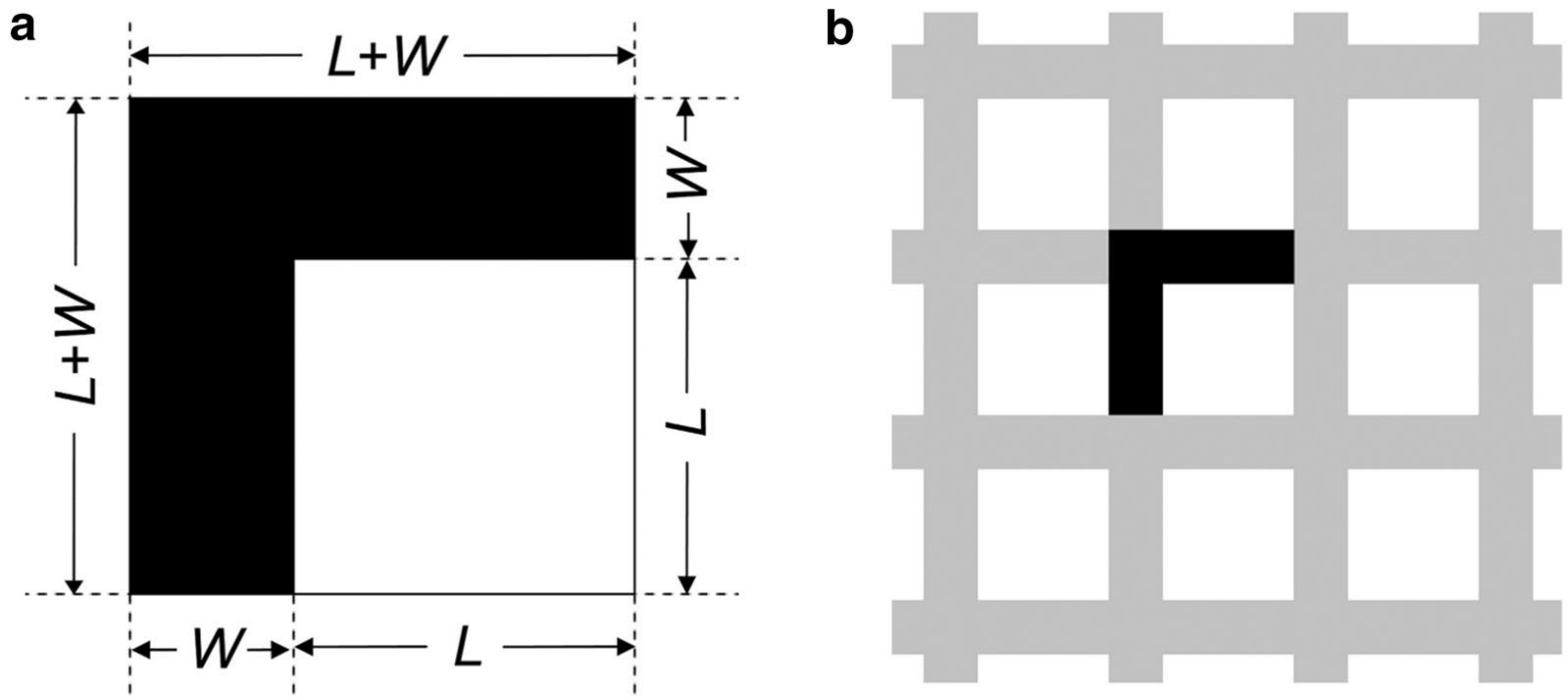
Design of (**a**) the basic unit for (**b**) a model canopy (gap fraction = 0.5). *L* is the side length of square openings. *W* is the separation width between square openings

Model canopies were constructed from opaque black adhesive vinyl sheet (20 cm by 21 cm) with square openings of different dimensions, stuck to clear acrylic plastic to eliminate distortion during handling. A range of gap fraction and SCB length was simulated through varying the side length (*L*) of square openings and the separation width (*W*) of these openings (Fig. 1, Table 1). Gaps in real canopies were simulated by square openings, while black grids were the analogue of canopy elements (Fig. 1b). Designed gap fraction (*GF*_d_) is equal to *L*^2^/(*L *+ *W*)^2^. For a desired combination of a given gap fraction and a side length of square opening, *W* can be acquired with the equation, $W = (L/\surd GF_{\text{d}} ) - L$ (Table 1).

**Table 1:** Designed gap fraction (*GF*_d_) and side length of square opening (*L*) of the 17 model canopies

Designed gap fraction (*GF*_d_)	Side length of square opening (*L*) (mm)			
	2.50	5.00	10.00	20.00
0.05	8.68, 0.08	17.36, 0.04	34.72, 0.02	–, –
0.10	5.41, 0.16	10.81, 0.08	21.62, 0.04	–, –
0.25	2.50, 0.40	5.00, 0.20	10.00, 0.10	20.00, 0.05
0.50	1.04, 0.80	2.07, 0.40	4.14, 0.20	8.28, 0.10
0.75	–, –	0.77, 0.60	1.55, 0.30	3.09, 0.15

The left and right values in each cell show the separation width between square openings (*W*; mm) and SCB length per unit area (*B*; mm mm^−2^) respectively. *GF*_d_ = *L*^2^/(*L *+ *W*)^2^. *B *= 4∙*L*/(*L *+ *W*)^2^

### Acquisition of CHPs

Three adapted CHP acquisition procedures were used to take photographs for the two-dimensional model canopies. First, model canopies backed with a piece of sand-scrubbed acrylic board (simulating the sky) was mounted at an end of a cylindrical pipe (internal diameter of 12.3 cm and length of 41 cm). The internal wall of this pipe was covered with black flannelette to reduce stray radiation. Second, a 28-watt ring fluorescent light (FLC-28, Falcon Eyes, Hong Kong, China) was placed behind the model sky to provide light for the model sky. A spot light meter (Spotmeter F, Konica Minolta, Tokyo, Japan) were used to make sure the illumination was even (illumination variation ≤ 0.2 stop) across the model sky and also measure the reference exposure from the model sky. Third, a Nikon D5000 camera (Tokyo, Japan) attached to Nikon AF-S ED 18–70 mm lens was mounted at the other end of the pipe. During the acquisition of model CHPs, the lens was always zoomed into 70 mm to reduce vignetting associated with wide view-angle lens. Through varying shutter speed but keeping aperture constant, CHPs for the 17 model canopies were taken with nine levels of exposure (− 3, − 2, − 1, 0, + 1, + 2, + 3, + 4 and + 5 stop) compensated from the reference exposure.

On 12 and 13 of August 2011, real CHPs were acquired in a forest about 500 m east of the Nanjenshan Research Station (22°05′05″N, 120°50′07″E) of Taiwan. *Schefflera octophylla*, *Psychotria rubra*, *Castanopsis cuspidata* var. *carlesii* and *Illicium arborescens* were the most dominant tree species in this forest (Chao et al. 2010). CHPs were acquired with a 4.5 mm F2.8 circular fisheye lens (Sigma Corp., Kanagawa, Japan) mounted on the same Nikon D5000 camera which was used for the acquisition of model CHPs. During the acquisition of CHPs, the camera was mounted on a tripod to keep it 1 m above ground and reduce camera shake. To eliminate focusing failure associated with automatic focusing, the focus of the fisheye lens was set to a constant distance, allowing that canopy elements more than 0.2 m away from the lens were included in the depth of field. Because CHPs acquired with the popular under-canopy exposure method (exposure of CHPs measured under canopy) tend to be overexposed (Chen et al. 1991; Zhang et al. 2005), we mainly examined the effects of overexposure on HP. Therefore, real CHPs were acquired with up to + 5 stop compensated from the reference exposure. Seven levels of exposure compensation were manipulated (− 1, 0, + 1, + 2, + 3, + 4 and + 5 stop) for every of the 46 photographing locations and totally 322 CHPs were acquired. Before CHP acquisition for every location, the exposure for CHPs was measured with a Nikon D300 camera following the above-canopy exposure method suggested by Zhang et al. (2005). This exposure-measuring camera was placed in an open area not farther than 0.5 km away from the CHP acquisition locations. This camera was attached with a Nikon AF-S ED 18–70 mm lens and mounted on a tripod. The lens was pointed to the sky zenith and zoom out to 18 mm to cover most of the sky area. The desired aperture for exposure compensation of + 5 stop was obtained with the following settings: shutter-priority of camera mode, ISO 1600 of light sensitivity, 1/30 s of shutter speed and + 5 stop of exposure compensation. Using this aperture coupled with the same setting of light sensitivity and shutter speed on the CHP-acquisition camera could acquire CHPs with exposure compensations of + 5 stop. CHPs of exposure compensations of + 4, + 3, + 2, + 1, 0 and − 1 stop were acquired by keeping aperture constant but using shutter speeds of 1/60, 1/125, 1/250, 1/500, 1/1000 and 1/2000 s respectively. During CHP acquisition, all the following settings remained constant, including image format (Jpeg), image size (4288 × 2848 pixels), image quality (fine), metering mode (matrix), sensitivity to light (ISO 1600), colour mode and white balance (cloudy sky).

### Quantifying the grey-pixel fraction and SCB length

The method of Leblanc et al. (2005) was modified to quantify the grey-pixel fraction in our CHPs. Sharp turns of curves in the greyness histograms of CHPs were used to visually identify the low threshold value separating canopy and grey pixels and the high threshold value separating sky and grey pixels. Our method differed from that of Leblanc et al. (2005) in that the scale of the y axis (pixel fraction) of our histograms was linear rather than logarithmic. The grey-pixel fraction was the proportion of pixels with digit numbers between the low and high threshold values. Macfarlane (2011) has proposed an automatic method (two-corner method) using two humps of greyness histograms to identify the low and high threshold values. This method was not used in the present study because many greyness histograms of our CHPs had only one hump due to overexposure or low canopy gap fraction.

When the side length (*L*) and separation width (*W*) of square openings are given, the SCB length per unit area (*B*) is equal to 4∙*L*/(*L *+ *W*)^2^ (Fig. 1, Table 1). Because there is no direct method to assess the SCB length for real canopies, the MPZ length was therefore measured as an approximation of the SCB length. The first step of measuring the MPZ length was to transform real CHPs into correctly-thresholded binary images by following the exposure-corresponding method proposed by Song et al. (2014). Pixels of MPZs in binary images were then identified with the function of “Find edges” of ImageJ version 1.43 (Abramoff et al. 2004). This function uses the Sobel edge detector which lays out a three-by-three convolution kernel vertically and the other horizontally relative to the pixel grid to detect sharp greyness changes for two dimensional images (Ferreira and Rasband 2012). The MPZ length of a CHP is the proportion of pixels in MPZs (hereafter, pixel fraction of MPZ). Analyses showed that the MPZ length obtained through this method was a close approximation of SCB length per unit area (Additional file 1: Figure S1).

### Assessing HP inaccuracy associated with the ETM

Because exposure and thresholding of CHPs can have both independent and interacting effects on HP accuracy (Song et al. 2014), these two processes should be examined together. A uniform basis for comparison of CHP exposure and thresholding effects was achieved by using two parameters, exposure manipulation and thresholding manipulation. Exposure manipulation was the extent of exposure deviating from the reference exposure measured under an unobscured overcast sky. Thresholding manipulation was transformed from the grey-scale threshold value using the regression model established by Song et al. (2014), in which the relationship between exposure manipulation (*X*) and the optimal threshold value (*Y*) is *Y* = 260.542/(1 + e^(−*X*+1.422)/1.160^). This model determines the optimal threshold values for CHPs exposed with specific exposure manipulations. If a CHP is analysed with a threshold value which is the optimal threshold value for CHPs acquired with exposure manipulation of *S* stop, this CHP is analysed with thresholding manipulation of *S* stop. For example, the optimal threshold value for CHPs acquired with exposure manipulation of + 3 stop is 202. When a CHP is analysed with a threshold value of 202, its thresholding manipulation is + 3 stop. The ETM extent can be quantified by subtracting the thresholding manipulation from the exposure manipulation. When the ETM extent of a CHP is 0 stop, that means this CHP is analysed with its optimal threshold value and it can provide a correct estimate for HP. Positive ETM extents indicate that CHPs are overexposed or analysed with threshold values that are too low, so that gap fraction is overestimated. Negative ETM extents underestimate gap fraction, as a result of underexposing CHPs or analysing CHPs with threshold values that are too high.

Because gap fraction is the HP estimation with the most direct connection between image and estimate (Jonckheere et al. 2005), we used gap fraction to evaluate the influence of grey pixels on HP inaccuracy. Prior to image analysis, the colour model and real CHPs were transformed into 8-bit greyscale images first. For model canopies, the correct gap fraction was obtained by analysing scanned images of model canopies (*GF*_s_) (details in Additional file 1: Text S1). To obtain photographed gap fraction (*GF*_p_) estimated with different thresholding manipulations, the amount of pixels for each digit number was obtained for every model CHP with the function of “Histogram” of ImageJ version 1.43 (Abramoff et al. 2004). Photographed gap fraction for the threshold value *i* (*GF*_p*i*_) was obtained through dividing the total number of pixels with digit numbers ≥ *i* by the total number of pixels in the circular images of CHPs. Using the relationship between optimal threshold value and threshold manipulation, we could obtain gap fraction under different thresholding manipulations for every model CHP. The estimation inaccuracy of model CHPs (*I*_m_) was obtained by subtracting the correct gap fraction from the photographed gap fraction (*I*_m_ = *GF*_p _− *GF*_s_).

For real canopies, the correct gap fraction was obtained by processing CHPs with matched exposure manipulation and thresholding manipulation (*GF*_match_). The estimation inaccuracy of real CHPs (*I*_r_) was obtained by subtracting the correct gap fraction from the gap fraction obtained with a mismatch combination of exposure and thresholding manipulations (*GF*_mismatch_) (*I*_r_ = *GF*_mismatch _− *GF*_match_). This method for obtaining correct estimates can ensure that the ETM is the only source of estimation inaccuracy. If correct estimates are obtained with other often-used approaches (such as diffuse light transmittance obtained with quantum sensors), additional sources of estimation inaccuracy (e.g. differences between instruments) may obscure the relationship between HP inaccuracy and the ETM.

### Data analysis

The *F*-test was used to evaluate the slope difference of regression lines with zero intercept, including regression lines between the SCB length and HP inaccuracy, and between the MPZ length and HP inaccuracy. It is assumed that there are totally *n* observations in *k* groups. The total sum of square (*SST*) is the sum of square for residuals between observations and the grand regression line. The sum of square within groups (*SSW*) is the sum of square for residuals between observations in the same groups and the group-specific regression lines. The sum of square between groups (*SSB*) is obtained by subtracting SSW from SST (Sokal and Rohlf 1995). The degree of freedom within groups (*DFW*) and between groups (*DFB*) is equal to *n *− *k* and *k *− 1 respectively (Sokal and Rohlf 1995). *F* values are calculated where.

1 $$F\,{\text{value }} = \frac{\text{Mean square among groups}}{\text{Mean square within groups}} = \frac{SSB/DFB}{SSW/DFW}$$

Data analyses and significance tests in the present study were conducted with Microsoft Excel 2010 (Redmond, Washington, US).

## Results

**Fig. 2:**
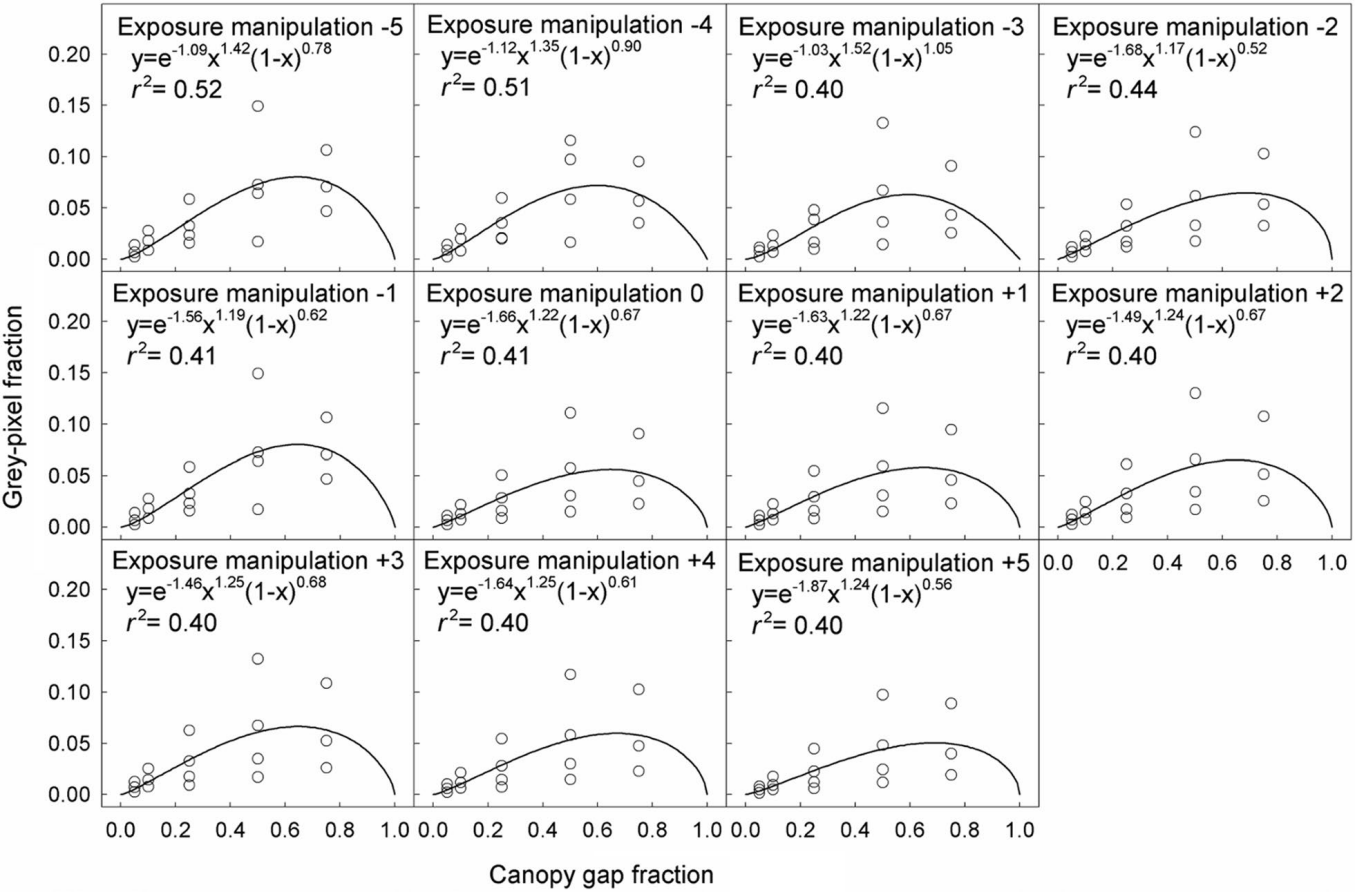
Relationships between the grey-pixel fraction and canopy gap fraction in model CHPs. The unit of exposure manipulation is stop

**Fig. 3:**
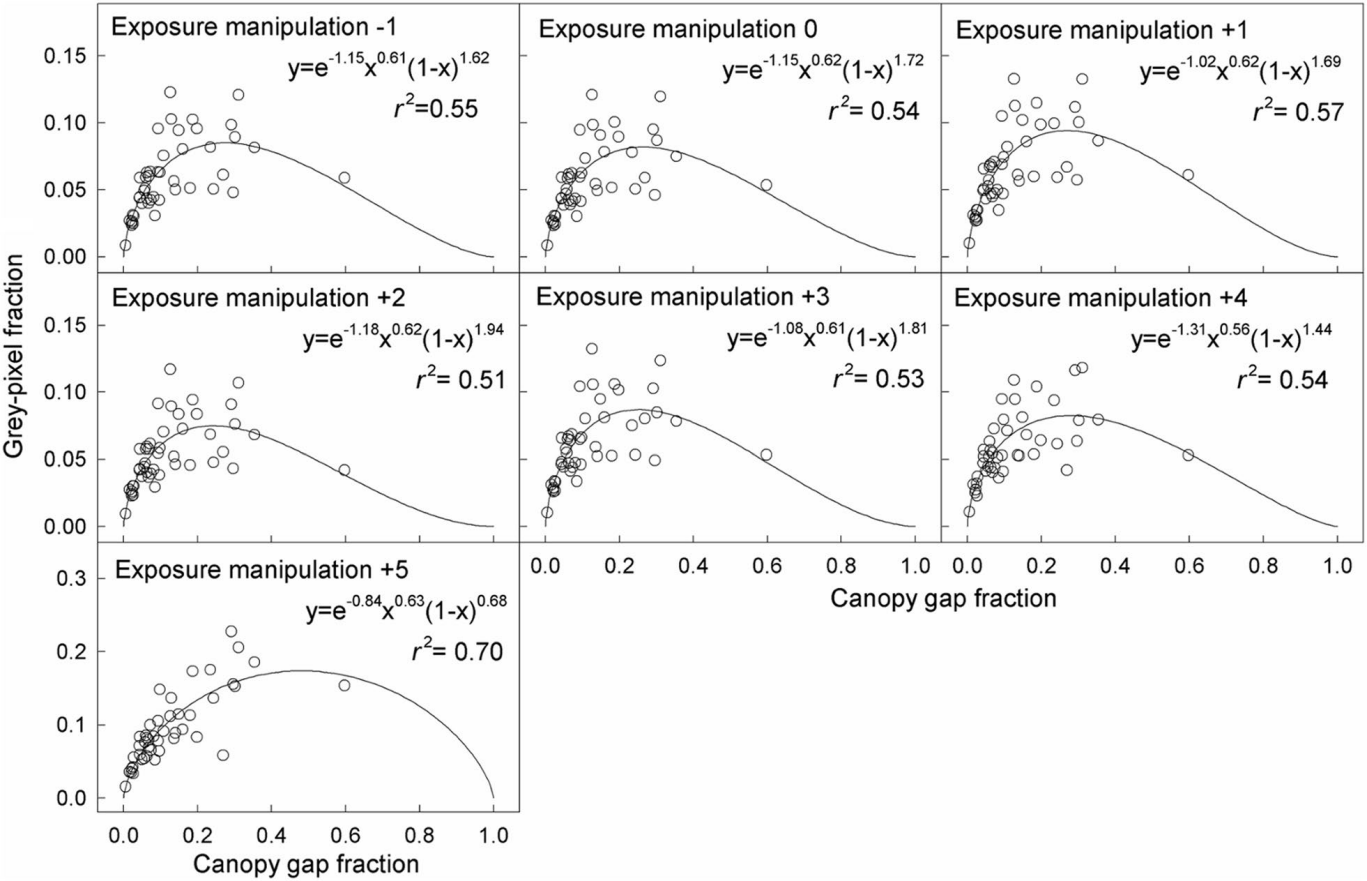
Relationships between the grey-pixel fraction and canopy gap fraction in real CHPs. The unit of exposure manipulation is stop

**Fig. 4:**
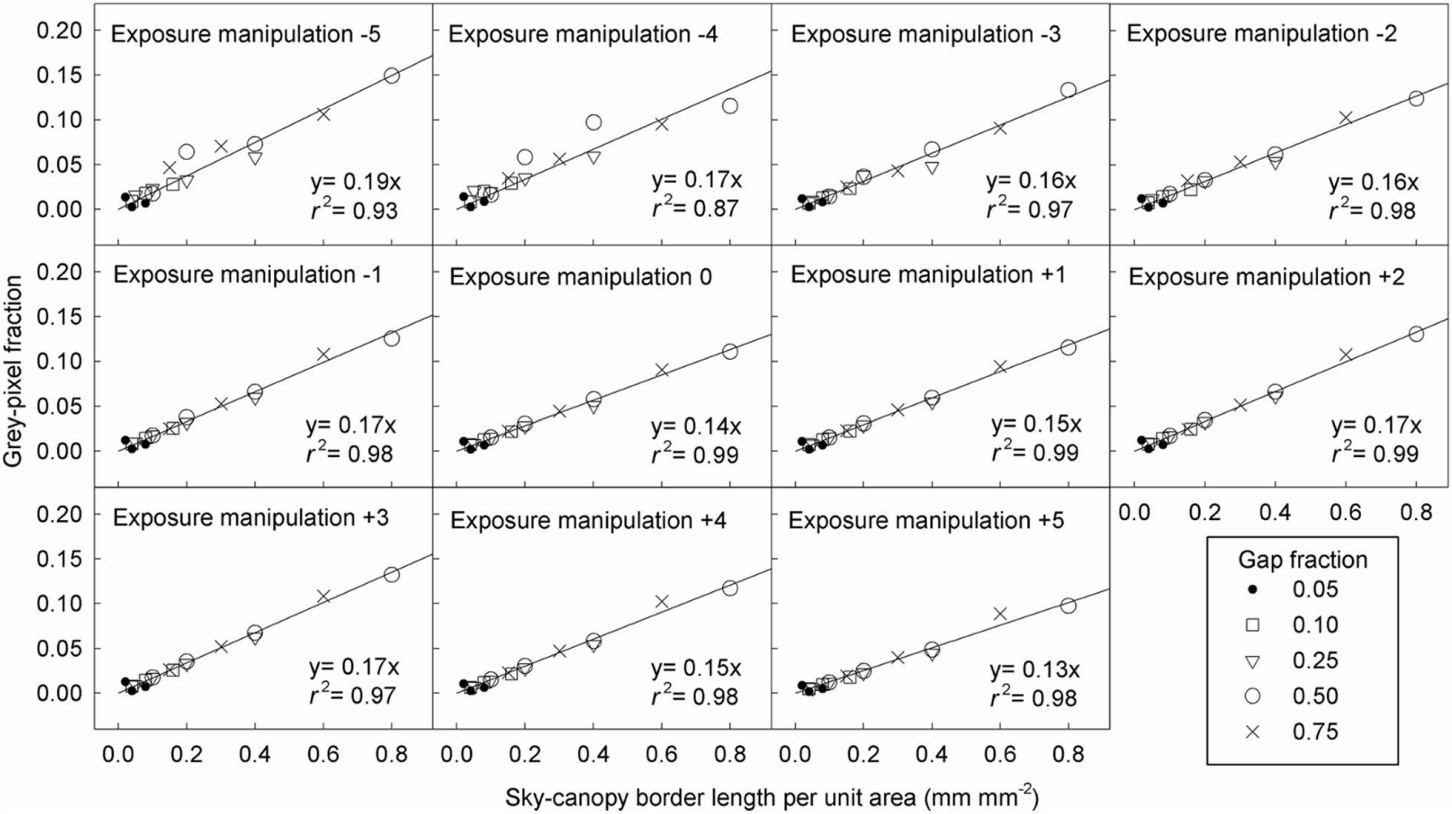
Relationships between the grey-pixel fraction and the SCB length of model canopies in model CHPs. The unit of the exposure manipulation is stop

**Fig. 5:**
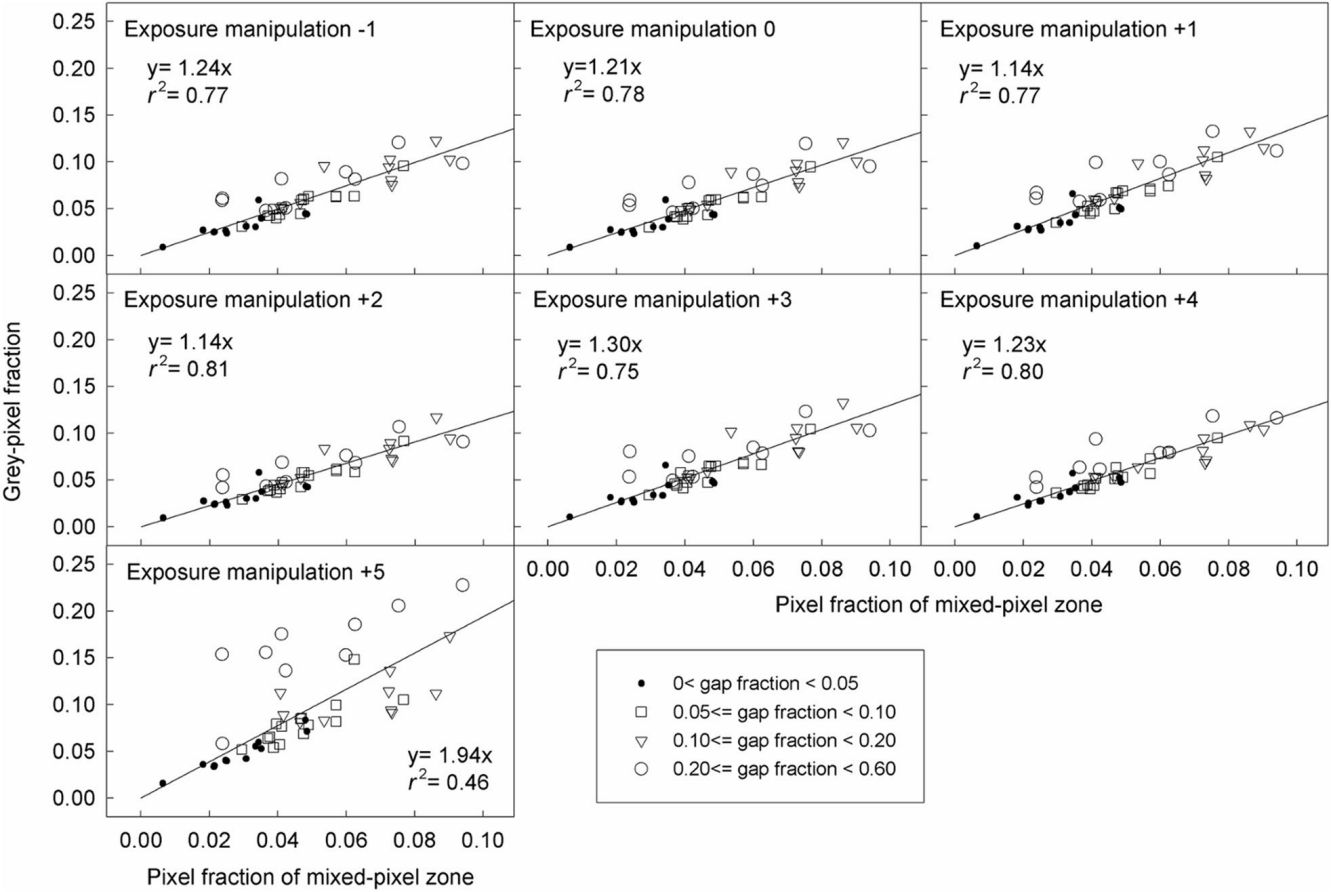
Relationships between the grey-pixel fraction and the mixed-pixel zone length in real CHPs. The pixel fraction of mixed-pixel zone is an approximation of the SCB length. The unit of the exposure manipulation is stop

The relationship between the grey-pixel fraction and canopy gap fraction was hump-shaped (Figs. 2 and 3). The coefficients of determination (*r*^2^) for fitting this relationship with linear models were lower than with hump-shaped models (Additional file 1: Figures S2 and S3). In contrast, the grey-pixel fraction was linearly related to the SCB length and MPZ length, but independent of canopy gap fraction (Figs. 4 and 5).

**Fig. 6:**
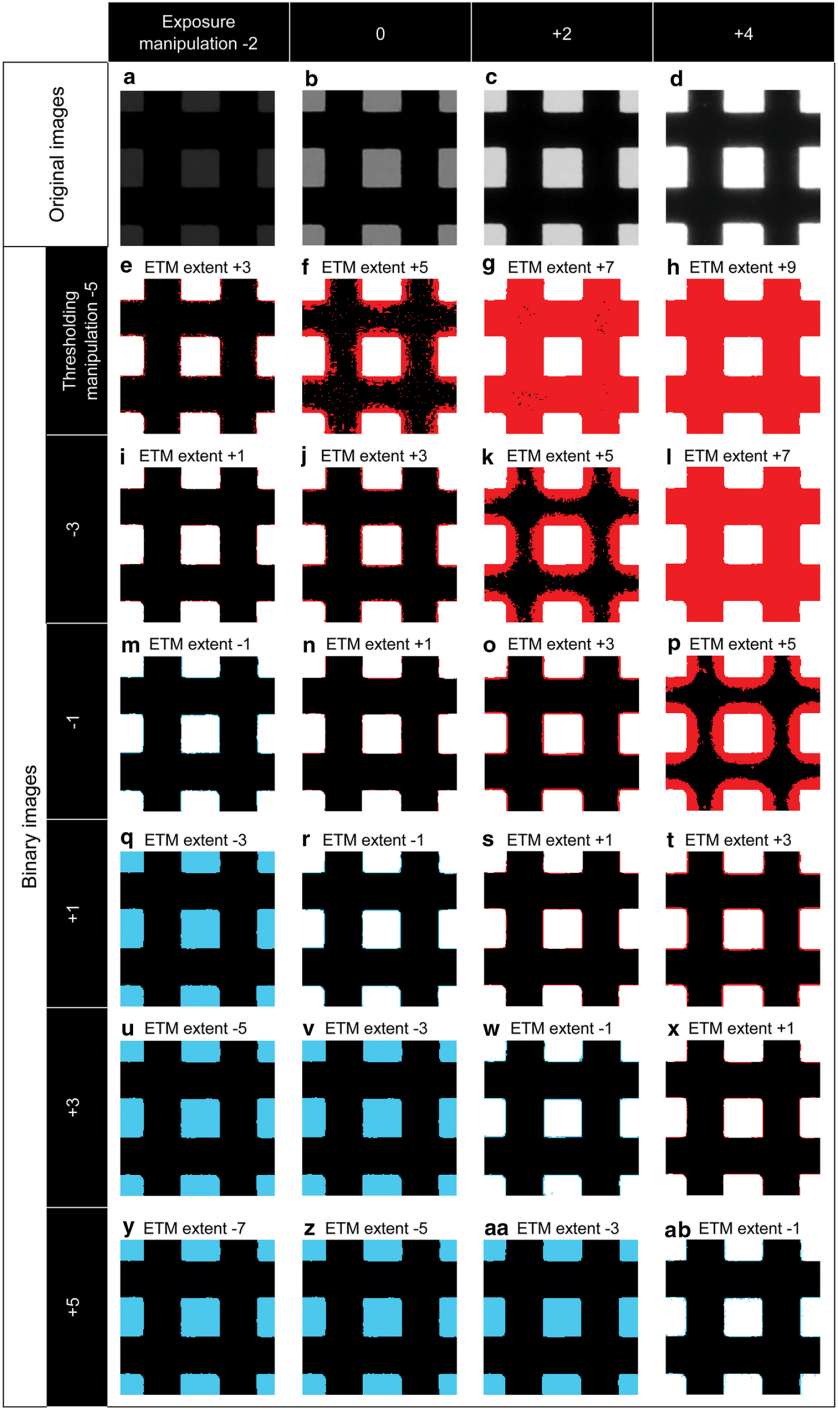
Cropped binary images of model CHPs with the ETM extents ranging from − 7 to + 9 stop. (**a**)–(**d**) are the original images of model CHPs acquired with exposure manipulation of − 2, 0, + 2 and + 4 stop. (**e**)–(**ab**) are the binary images obtained with combinations of 4 exposure manipulations (− 2, 0, + 2, and + 4 stop) and 6 thresholding manipulations (− 5, − 3, − 1, + 1, + 3, and + 5 stop). Canopy and grey pixels which are misclassified as sky pixels are shown in red; sky and grey pixels which are misclassified as canopy pixels are blue; sky and grey pixels which are correctly classified as sky are white; canopy and grey pixels which are correctly classified as canopy are black. These CHPs were taken for a model canopy with gap fraction of 0.25 and SCB length per unit area of 0.4 mm mm^−1^ (i.e. side length of square opening of 2.5 mm). The diameter of the original circular images of these model CHPs is 2098 pixels, and the image dimension of cropped binary images shown in this figure is 177 by 177 pixels. Greyness histograms of these CHPs presented in this figure are shown in Fig. 7. The unit of the exposure manipulation, thresholding manipulation and ETM extent is stop

Panels in the same columns of Fig. 6 show binary images which exposure manipulation is kept constant but thresholding manipulation is varied. Panels in the same rows of Fig. 6 shows binary images which thresholding manipulation is kept constant but exposure manipulation is varied. As long as the ETM extent is kept constant, even though the exposure manipulation and thresholding manipulation paired together vary, the amount and spatial distribution of misclassified pixels are almost identical (e.g. Fig. 6e, j, o, t, m, r, w, ab). This indicates that the amount of misclassified pixels is not determined solely by either exposure manipulation or thresholding manipulation, but the ETM extent (Fig. 6). When the ETM extent is lower than − 1 stop, all pixels of model CHPs are classified as canopy pixels (Fig. 6q, u, v, y, z, aa). When the ETM extent ranges from − 1 to + 3 stop, those misclassified pixels were all located on where sky and canopy elements meet (Fig. 6e, i, j, m, n, o, r, s, t, w, x, ab). That is, misclassified pixels were those pixels recording SCBs. Once the ETM extent reached + 5 stop, a great proportion of canopy pixels (pixels of grids) are misclassified as sky pixels (Fig. 6f, k, p). All pixels are classified as sky pixels, when the ETM extent is equal to or greater than + 7 stop (Fig. 6g, h, l).

**Fig. 7:**
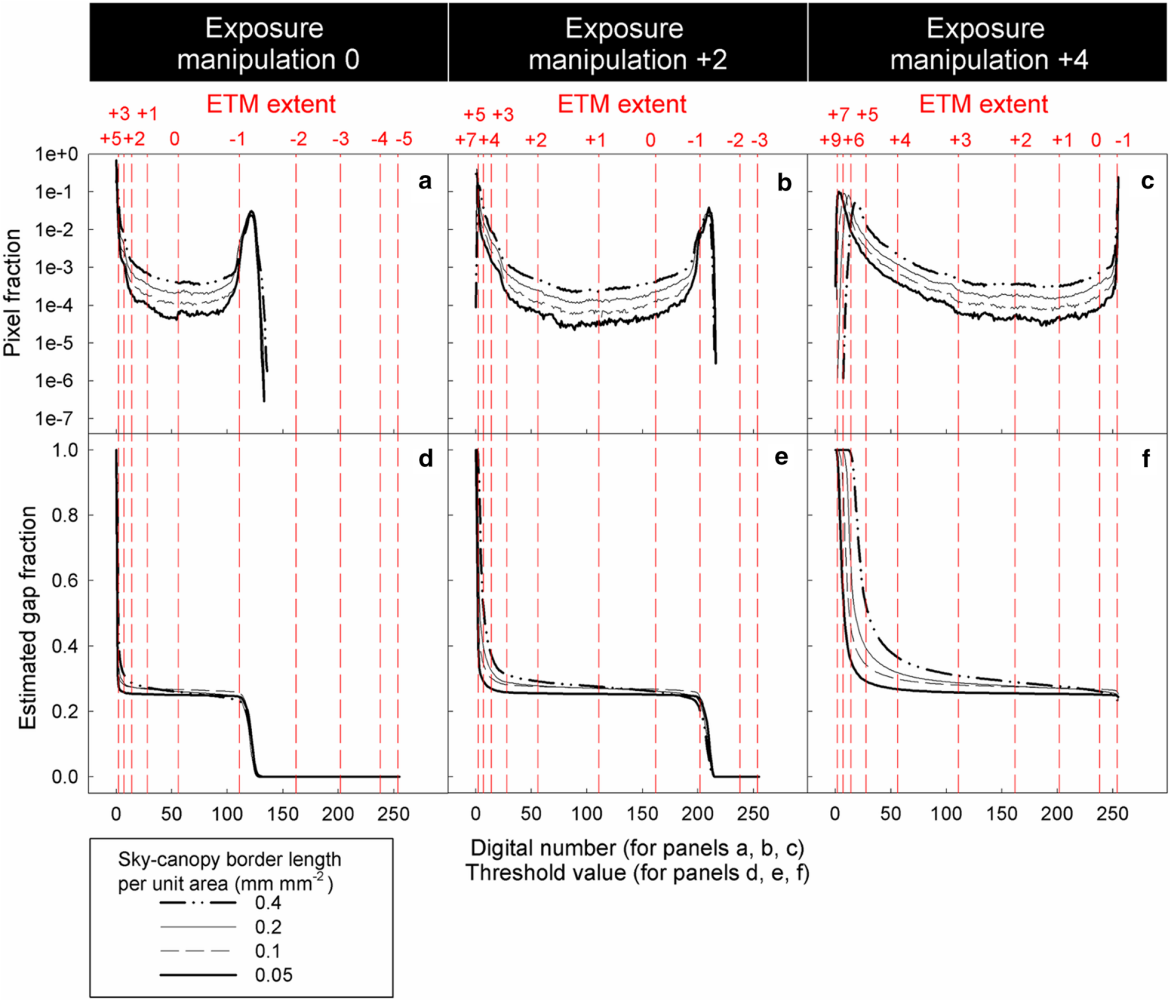
**a**–**c** Frequency distribution of pixel fraction along the greyness gradient of model CHPs, and **d**–**f** estimated gap fraction under different ETM extents. In **a**–**c**, grey pixels are those in between the humps of canopy and sky pixels. The figure is derived from CHPs of four model canopies with the same designed gap fraction of 0.25 and SCB length per unit area ranging from 0.05 to 0.4 mm mm^−1^. Note log scale used on y-axis of panels **a**–**c**. The unit of the exposure manipulation, thresholding manipulation and ETM extent is stop

**Fig. 8:**
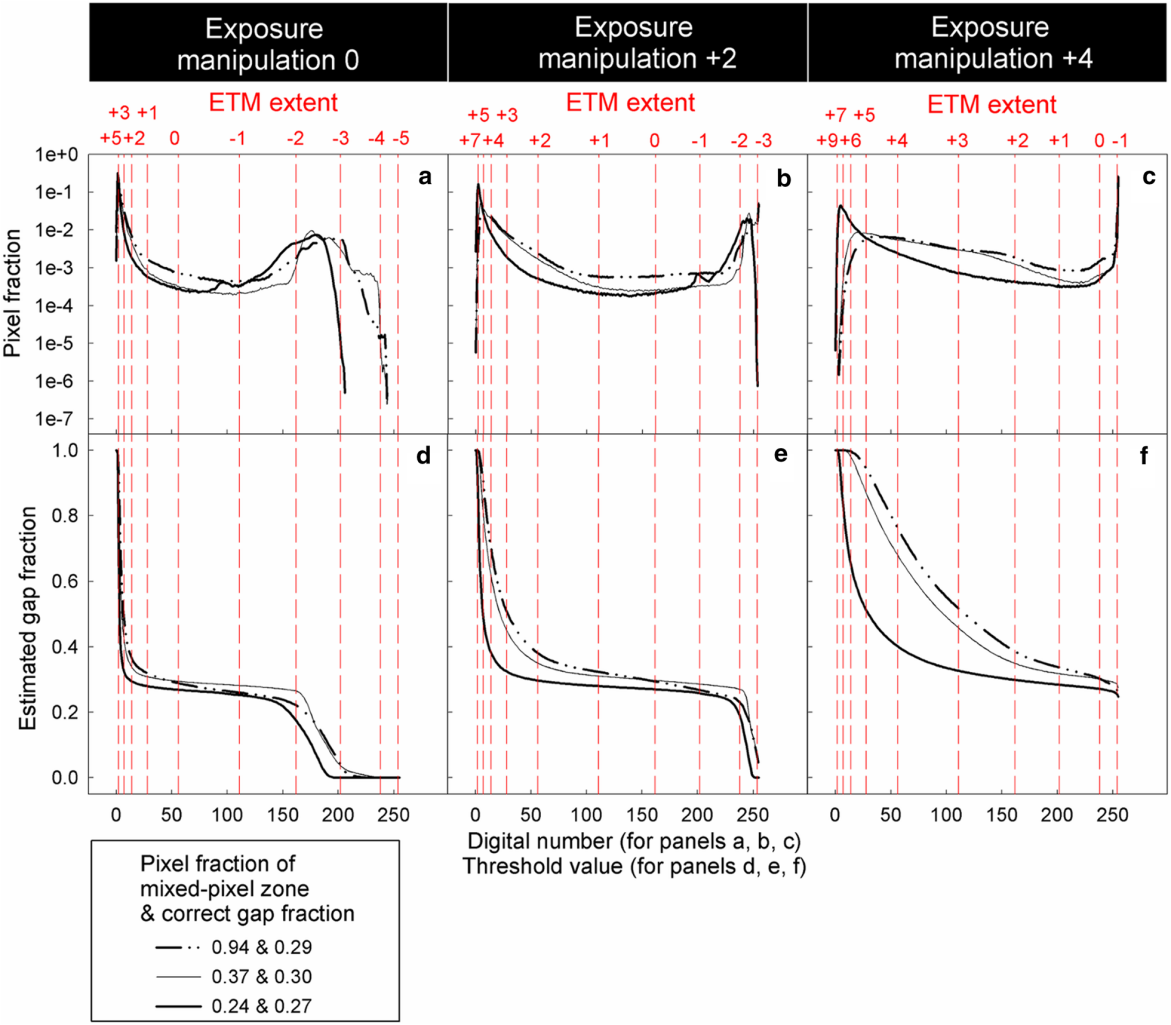
**a**–**c** Frequency distribution of pixel fraction along the greyness gradient of real CHPs, and **d**–**f** estimated gap fraction under different ETM extents. The figure is derived from CHPs of three real canopies with similar gap fraction (0.29, 0.30 and 0.27), with proportion pixel of mixed-pixel zone (i.e. mixed-pixel zone length) of 0.94, 0.37 and 0.24, respectively. Note log scale used on y-axis in panels **a**–**c**. The unit of the exposure manipulation, thresholding manipulation and ETM extent is stop

Panels a–c in Figs. 7 and 8 show greyness histograms (the frequency distribution of pixel fraction along the greyness gradient) of model and real CHPs respectively. In Figs. 7a–c, 8a–c, every curve has two humps. Humps at lower digital numbers are contributed by canopy pixels, whereas humps at higher digital numbers are contributed by sky pixels. Pixels in between these two humps are the grey pixels. The grey pixel fraction increases with increasing SCB length (Fig. 7a–c) and MPZ length (Fig. 8a–c). Panels d–f in Figs. 7 and 8 show gap fraction estimated with threshold values from 0 to 255. As long as threshold values within the greyness range of grey pixels (ETM extent from − 1 to + 3 stop) are used for thresholding (i.e. only grey pixels are misclassified), estimated gap fraction increases with ETM extents (Figs. 7d–f and 8d–f).

**Fig. 9:**
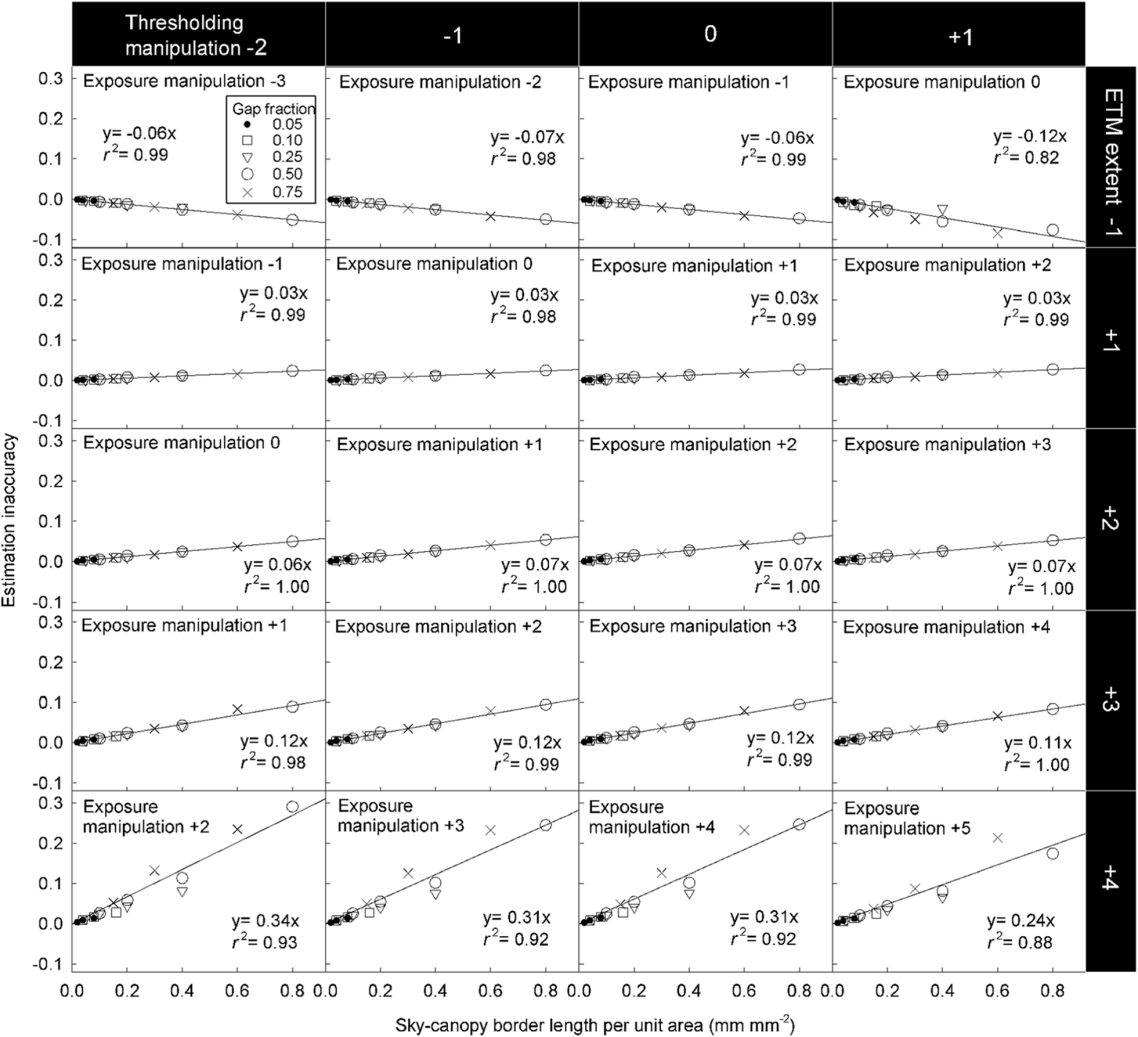
Effects of the SCB length, canopy gap fraction and the ETM extent on the HP estimation derived from model CHPs of 17 model canopies. The unit of the exposure manipulation, thresholding manipulation and ETM extent is stop

**Fig. 10:**
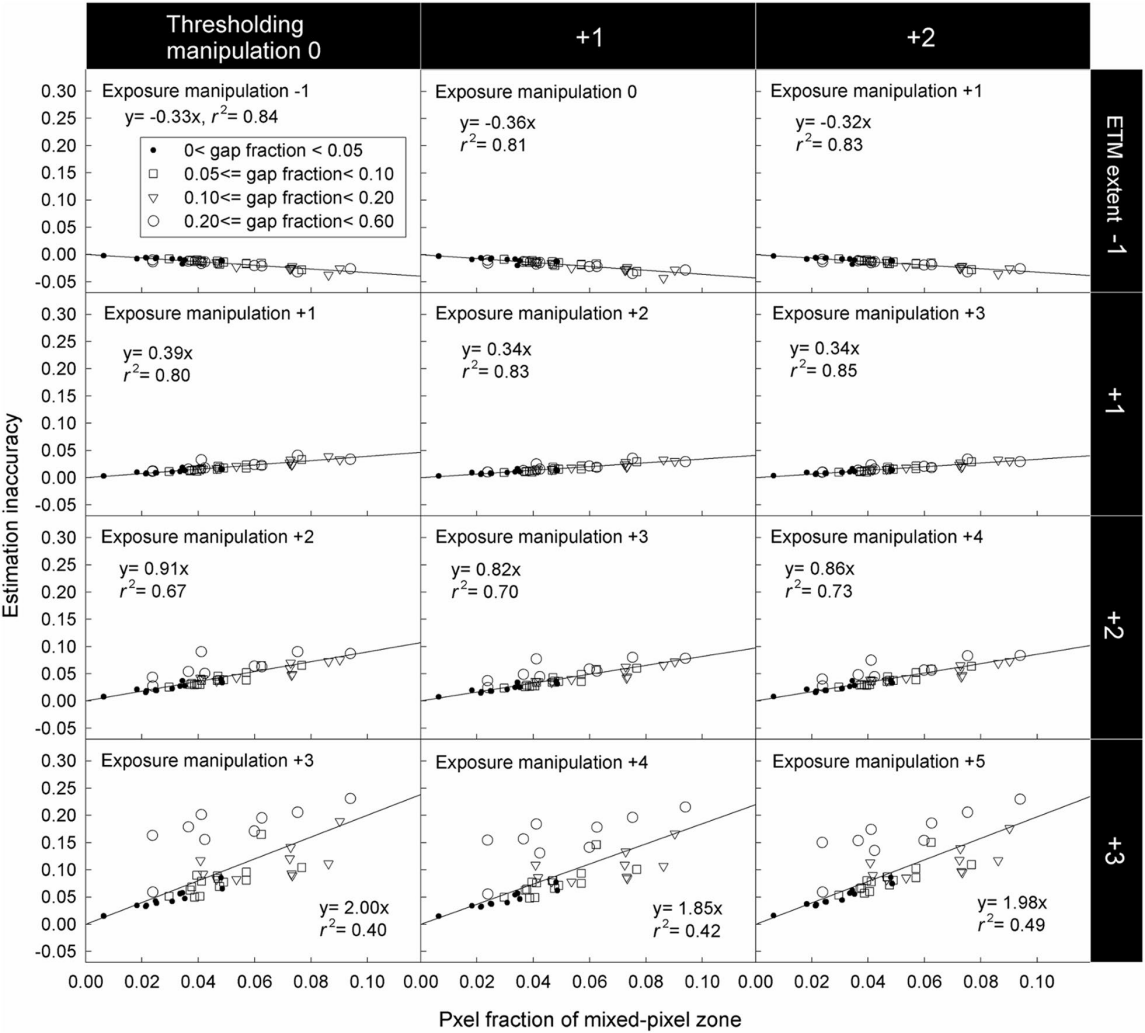
Effects of the mixed-pixel zone length (i.e. pixel fraction of mixed-pixel zone), canopy gap fraction and the ETM extent on the HP estimation derived from real CHPs taken in 46 locations. The unit of the exposure manipulation, thresholding manipulation and ETM extent is stop

As long as only grey pixels are misclassified (i.e. the ETM extent is not zero and ranges from − 1 to + 3 stop), HP inaccuracy is positively related to the SCB length (Fig. 9) and MPZ length (Fig. 10). However, the HP inaccuracy is independent of canopy gap fraction for both model and real CHPs (Figs. 9 and 10). Significant tests showed that slopes of most regression lines with the same ETM extents (panels within the same rows in Figs. 9 and 10) differed significant in Fig. 9 (Table 2). This was a surprising result especially for regression lines within the same ETM extents of + 1 to + 3 stop in Fig. 9, because their slopes were almost identical. This result was attributed to that our control on the light intensity of the light source was not precise enough during the acquisition of model CHPs. For the detailed explanation, please see Additional file 1: Text S2. In panels within the same columns in Figs. 9 and 10, the slopes of regression lines approximately doubled with each stop increase in the ETM extent, indicating a positive linear relationship between HP inaccuracy and the ETM extent in both model and real CHPs.

**Table 2:** Significant tests for slopes of regression models with the same ETM extents

Type of CHPs	ETM extent	DFW	DFB	*F* value	*P* value
Model CHPs	− 1	64	3	34.899	0.007
	+ 1	64	3	12.444	0.030
	+ 2	64	3	32.819	0.007
	+ 3	64	3	9.870	0.041
	+ 4	64	3	5.244	0.098
	− 1	134	2	5.099	0.178
Real CHPs	+ 1	134	2	9.742	0.097
	+ 2	134	2	2.087	0.380
	+ 3	134	2	0.561	0.828

These regression model are shown in Figs. 9 and 10

*CHP* canopy hemispherical photograph, *DFW* degree of freedom within groups, *DFB* degree of freedom between groups

## Discussion

**Fig. 11:**
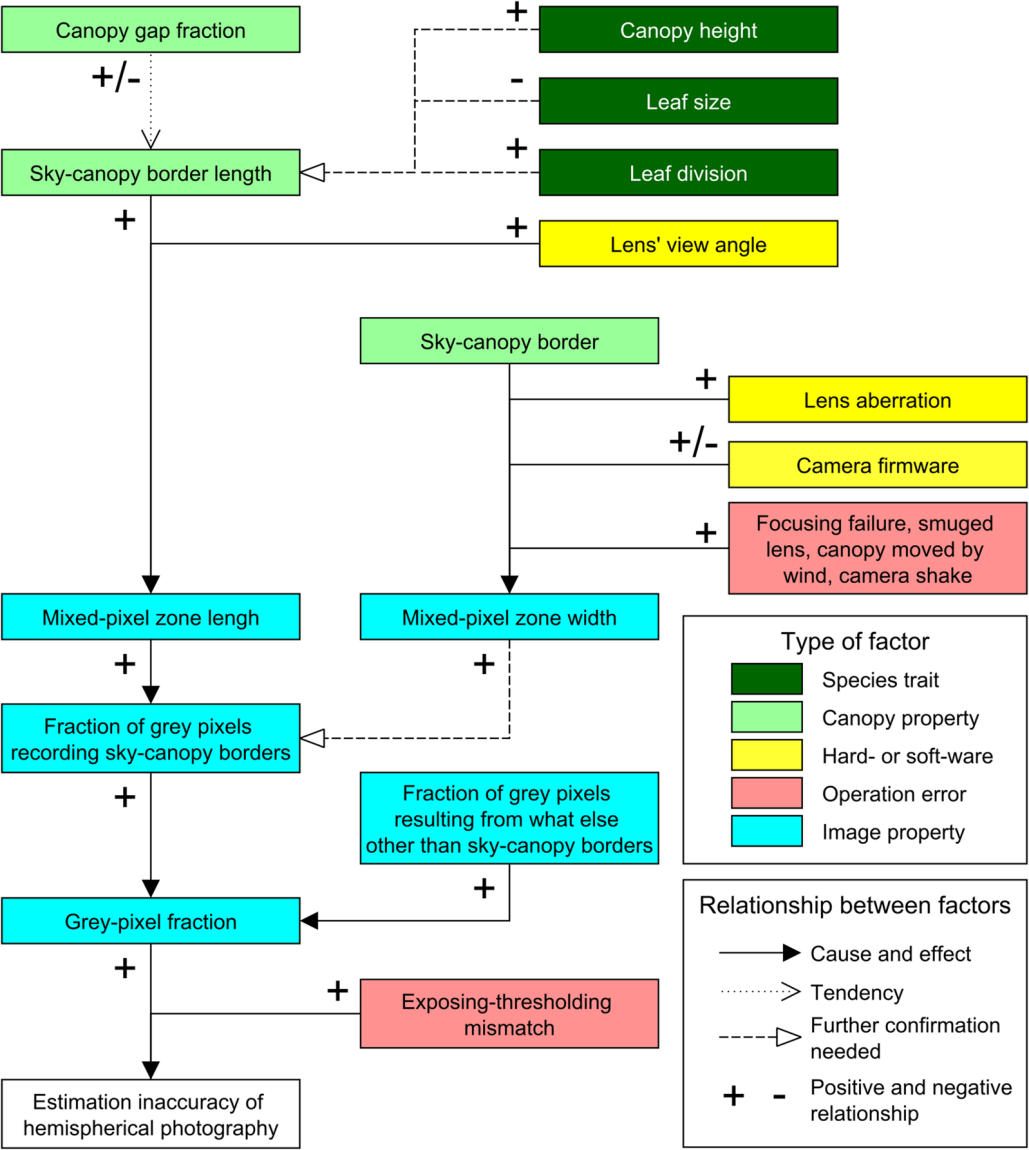
Relationships between the estimation inaccuracy of HP and its influential factors. Minus and plus signs indicate negative and positive relationships, respectively

For ease of understanding, the mechanisms of how HP accuracy for canopy gap fraction is affected by canopy properties (gap fraction and SCB length), exposure and thresholding errors, and other factors are illustrated by a schematic diagram (Fig. 11).

### Relationships between canopy properties, the grey-pixel fraction, the ETM and HP inaccuracy

The relationship between canopy gap fraction and grey-pixel fraction is actually mediated by the SCB length (Fig. 11). Although the grey-pixel fraction was related with canopy gap fraction (Figs. 2 and 3), this relationship became negligible if the SCB length (Fig. 4) or MPZ length (Fig. 5) was included. Moreover, in the model canopy system, the SCB length and gap fraction could be varied independently (Table 1, Additional file 1: Figure S4). These results indicated that the relationship between gap fraction and the SCB length in Figs. 2 and 3 is a tendency rather than a cause-and-effect relationship, so is the relationship between gap fraction and the grey-pixel fraction (Fig. 11).

The relationship between canopy gap fraction and the grey-pixel fraction is hump-shaped (Figs. 2 and 3) rather than linear (as reported by Macfarlane (2011)). When canopy gap fraction is zero or one, because of the absence of either sky or canopy elements, the SCB length has to be zero, and so do the MPZ length in CHPs. Consequently, if CHPs are sorted with gap fraction from zero to slightly higher values, the MPZ lengths of these CHPs increase from zero to greater values. If CHPs are sorted with gap fraction from one to slightly lower values, the MPZ lengths of CHPs also increase from zero to greater values. In summary, the relationship between canopy gap fraction and the grey-pixel fraction has to be hump-shaped. The study of Macfarlane (2011) was conducted in forests with relatively high canopy gap fraction (approximately ranging from 0.15 to 0.6), he therefore observed the negative relationship near the higher end of the gap fraction gradient.

The mixed-pixel fraction is a function of not only the length but also the width of MPZs (Fig. 11). In images, the width of edges between two different objects (i.e. the MPZ width in the present study) decreases with the increase of image sharpness (Allen and Triantaphillidou 2011; Smith 1997) (Fig. 11). As mentioned in the introduction, even with optically-perfect lens, MPZs are at least as wide as one pixel due to the grid pixel system of digital images (Fisher 1997). Because image resolution of the image capturing devices in many up-to-date digital cameras exceeds that of lens (https://www.dxomark.com/Reviews/Looking-for-new-photo-gear-DxOMark-s-Perceptual-Megapixel-can-help-you, 20th April, 2017), blurred SCBs caused by lens imperfections are recorded in details, resulting in that MPZs are even wider. In addition, image sharpness is also influenced by the image-processing algorithms of camera firmware and operation errors during image acquisition (Allen and Triantaphillidou 2011) (Fig. 11). Effects of lens and firmware can be controlled by using the same hard- and soft-ware respectively. In contrast, effects of operations during image acquisition can vary greatly shot by shot. When taking CHPs, HP users should minimise the commonly-encountered operation errors, including focusing failure, smudged lens, canopy movement caused by wind and camera shake (Fig. 11).

In a CHP, the relationships among the overall grey-pixel fraction (*F*), the SCB length (*B*), image sharpness (*S*) and the fraction of grey pixels recording what else other than SCBs (*G*) can be summarised by Eq. 2. In practice, image sharpness of CHPs can be quantified with the 10–90% edge response distance, which is a parameter measuring the distance required for the edge response rising from 10 to 90% of the greyscale amplitude (Smith 1997).

2 $$F = B/S + G$$

Even in real CHPs, grey pixels consist mainly of pixels recording SCBs. Due to the model canopy properties mentioned in the introduction, grey pixels recording what else other than SCBs (*G*) is eliminated from model CHPs, giving rise to almost perfectly linear relationships between the overall grey-pixel fraction (*F*) and the SCB length (*B*) (Fig. 4, Eq. 2). Because bright canopy elements and dark skies are inevitable properties of real CHPs (Rich 1990), *G* must be higher in real CHPs than in model CHPs. Consequently, coefficients of determination in Fig. 5 are lower than those in Fig. 4. In other words, high *G* weakens the linear relationship between *F* and *B* (Eq. 2). Nevertheless, except exposure manipulation of + 5 stop, all coefficients of determination in Fig. 5 were greater than 0.75, indicating that the majority of grey pixels in real CHPs are pixels recording SCBs. In other words, *G* in real CHPs is low enough so that the linear relationships between *F* and *B* are observed in Fig. 5. Exposure manipulation of + 5 stop raised the digit number of canopy elements to the level that many canopy pixels were recognised as grey pixels and in turn weakened the linear relationship between *F* and *B* (Fig. 5).

As long as only grey pixels are misclassified, Eq. 3 bellow can summarise the relationship between HP inaccuracy (*I*) and its influential factors (*M*, ETM extent; *F*, the overall grey-pixel fraction; *B*, the SCB length; *S*, image sharpness; *G*, the fraction of grey pixels resulting from what else other than the SCBs) in Figs. 4, 5, 9 and 10.

3 $$I = M \cdot F = M \, \cdot \left( {B/S + G} \right)$$

The linear relationship between the ETM extent and HP inaccuracy is applicable for all forest types. In addition to studies of Macfarlane et al. (2000) and Macfarlane (2011), this relationship has been implicitly reported in Fig. 4b of Hwang (2016), Fig. 4 of Macfarlane et al. (2014), Fig. 3 of Thimonier et al. (2010) and Figs. 4 and 5 of Zhang et al. (2005). Figures of these studies which took CHPs for model canopies or real forests with different species composition all showed that each stop of exposure variation introduces approximately the same magnitude of HP inaccuracy. In the present study, despite the fact that the geometry of model and real canopies differed substantially, linear relationships between the ETM extent and HP inaccuracy were observed in both model and real CHPs (Figs. 9 and 10). Results of the previous and present studies indicated that this linear relationship is applicable to all forest types.

### Suggestions for operations and future studies of HP

The lack of precision on exposure and thresholding can lead to higher HP inaccuracy for images with long than short sky-canopy borders (Figs. 9 and 10). There is a need to know what kind of canopies are of long SCBs. Plants with taller canopies tend to have longer SCBs. When doubling the camera-to-canopy distance, the SCB length in captured images increases by a factor of two (Additional file 1: Figure S4), so do the grey-pixel fraction. In other words, the taller the canopies (i.e. the greater camera-to-canopy distance), the higher the grey-pixel fraction of captured CHPs. Therefore, when exposure manipulation was controlled, HP inaccuracy increased with the distance between model canopies and the camera (Fig. 4 of Macfarlane et al. (2014)). Trees with smaller leaf sizes and higher degrees of leaf division may have more openings and in turn longer SCBs in their canopies (Fig. 11). Further studies are needed to identify effects of leaf size and leaf division on the SCB length in acquired images.

The MPZ length has the potential to be a universal parameter, which enable researchers to quantify the effects of canopy properties which are influential to HP estimation and in turn carry out cross-study comparisons for HP studies. Most HP researchers are aware that HP estimation is likely to be affected by species- and site-specific canopy properties, so they described where CHPs were taken and species composition and structure (e.g. stem density, tree height) of vegetation (e.g. Hwang et al. 2016; Jonckheere et al. 2005; Zhang et al. 2005). Nevertheless, these attributes provide little insight into how and to what extent they may affect HP accuracy. Imperfect exposure and thresholding are among the most frequently encountered errors in HP (e.g. Jonckheere et al. 2005; Rich 1990; Zhang et al. 2005). In the presence of exposure and thresholding errors, the SCB length and MPZ length can ultimately change HP estimates (Figs. 9 and 10) through their effects on the grey-pixel fraction (Figs. 4, 5 and 11). This indicates that the MPZ length, a close approximation for the SCB length, can be used as a parameter to quantify effects of canopy properties on HP estimation. As mentioned previously, the SCB length is affected by at least one canopy property (i.e. canopy height (Additional file 1: Figure S4)). Most important, the SCB length can quantify effects of canopy properties which can affect HP estimation. To facilitate cross-study comparisons, the MPZ length of CHPs should be provided in studies which use or develop the technique of HP.

The SCB length in photographs can be reduced through using lens with a narrower view angle. Although SCB length is an unmanipulable canopy property, SCB length captured by photographs can vary with lens’ view angle (Additional file 1: Figure S4). Cover photography developed by Macfarlane et al. (2007) uses lens with view angle narrower than that of the fisheye lens to acquire photographs for LAI estimation. When taking photographs for the same canopies, the narrower the lens’ view angle, the smaller fraction of canopies is captured, as a result, the shorter SCBs are recorded in photographs (Additional file 1: Figure S4). Because the grey-pixel fraction, a function of the SCB length (Fig. 4), of cover photographs was lower than that of hemispherical photographs (Chianucci 2016; Macfarlane 2011), estimation derived from cover photographs are more accurate than that based on hemispherical photographs (Chianucci 2016).

For photographs with long SCBs, e.g. photographs captured with wide view-angle lenses (fisheye lens), accurate exposure and thresholding are the key to reduce negative effects of grey pixels on HP estimation. As mentioned in the introduction, it is infeasible to eliminate grey pixels which record SCBs. However, HP inaccuracy caused by the misclassification of grey pixels can be minimised through reducing the ETM extent (Fig. 11; Eq. 3). Although extra efforts needed to establish the relationship between exposure and thresholding in advance, the exposure-corresponding method (Song et al. 2014) is one of the few objective methods which can integrate processes of exposure and thresholding and in turn reduce the ETM extent. Individually carrying out exposure and thresholding with correct methods is another approach to achieve accurate exposure and thresholding. Song et al. (2014) showed that using auto-thresholding methods to analyse CHPs with exposure of one to two stop higher than the reference exposure measured from an unobscured overcast sky (the so-called above-canopy exposure method (Zhang et al. 2005)) can also provide accurate HP estimation. Compared with the exposure-corresponding method, the advantage of this approach is that there is no need to establish the relationship between exposure and thresholding.

Manual thresholding is not recommended for studies which need high HP accuracy (e.g. monitoring temporal dynamics of understorey light environment in the same locations). Our results showed that the misclassified pixels in binary images is hardly detectable even when the ETM extent is up to + 3 stop (Fig. 6e, j, o, t). For example, Fig. 6o shows the binary image which was acquired with exposure manipulation of + 2 stop (its optimal threshold value is 162 (Song et al. 2014)) and analysed with thresholding manipulation of − 1 stop (that is, thresholding value of 28 was used (Song et al. 2014)). Even though the threshold value mismatch is as much as 134 (i.e. the ETM extent is + 3), the presence of misclassified grey pixels is barely noticeable if they are not coloured in red (Fig. 6o). On the other hand, the amount of grey pixels can account for as much as 10–15% of the total pixel number in real CHPs (Fig. 5) (Macfarlane 2011). This circumstance coupled with the misclassification of the majority of grey pixels will result in considerable HP inaccuracy. For example, when the ETM extent is + 3 stop, for half (23 out of 46) of our real CHPs source locations, the estimated gap fraction is more than twice the correct gap fraction (Additional file 1: Figure S5). In summary, it is very likely that estimates for gap fraction already deviate too much from correct ones before the grey-pixel misclassification reaches to the visually-detectable extent. As mentioned previously, for studies which require high HP accuracy, auto-thresholding methods used with the above-canopy exposure method (Zhang et al. 2005) and the exposure-corresponding method (Song et al. 2014) are recommended.

For studies developing exposure and thresholding methods for HP, photographs with long SCBs (i.e. photograph which are acquired with wide view-angle lenses and record long SCB canopies) should be used. As mentioned above, in terms of LAI estimation, cover photographs outperform CHPs because the former contain fewer grey pixels recording SCBs (Chianucci 2016). Our results also showed that the shorter the SCBs, the lower the HP inaccuracy (Fig. 11; Eq. 3). In other words, low HP inaccuracy is the result of not only the good performance of exposure and thresholding methods, but also short SCBs in photographs used for performance evaluation. To ensure that the true performance of exposure and threshold methods is identified, their performance evaluation should be carried out with photographs with long SCBs rather than cover photographs.

## Conclusions

The present study aims to identify how canopy properties (gap fraction and SCB length) and errors of operation (exposure and thresholding) influence an image property (grey-pixel fraction) of CHPs and ultimately affect HP estimation for gap fraction. Grey pixels in CHPs include pixels recording darkened sky, bright canopy elements, sub-pixel canopy elements and SCBs. Our results showed that exposure and thresholding errors cause the misclassification of grey pixels in binary images and in turn result in the HP inaccuracy. Moreover, the grey-pixel fraction of CHPs is a linear function of the SCB length because the majority of grey pixels are those recording SCBs. As a result, HP inaccuracy increases linearly with the SCB length of CHPs once there are exposure and thresholding errors. The relationship between canopy gap fraction and the grey-pixel fraction is, in fact, mediated by the SCB length. Results also showed that the linear relationship between HP inaccuracy and the extent of exposure and thresholding errors is applicable to all forest types. Compared with hemispherical photographs, cover photographs which are acquired with narrow view-angle lenses can provide more accurate estimation for HP, because narrow view-angle lenses capture only a small fraction of canopies and in turn shortens the SCB length in photographs. Due to the fact that short SCBs in photographs and low levels of exposure and thresholding errors can both result in low HP inaccuracy, using photographs with long SCBs rather than cover photographs can ensure that the true performance of new exposure and thresholding methods for HP is identified.

## Additional file

**Additional file 1: Figure S1.** Relationship between the sky-canopy border length and mixed-pixel zone length in model canopy hemispherical photographs. **Figure S2.** Using linear models to fit the relationships between the grey pixel fraction and canopy gap fraction in model canopy hemispherical photographs. **Figure S3.** Using linear and second order polynomial models to fit the relationships between the grey pixel fraction and canopy gap fraction in model canopy hemispherical photographs. **Figure S4.** Relationships of canopy gap fraction, the camera-to-canopy distance and lens’ view angle to the sky-canopy border length. **Figure S5.** Effects of the exposure-thresholding mismatch extents (ETM extents) on estimated gap fraction. **Text S1.** The method for obtaining the correct gap fraction for model canopies. **Text S2.** The impreciseness of our control on the light intensity of the light source when taking photographs for model canopies.

## Abbreviations

CHP: canopy hemispherical photograph
ETM: exposure-thresholding mismatch
HP: hemispherical photography
MPZ: mixed-pixel zone
LAI: leaf area index
SCB: sky-canopy border
