# Supplementary material for: Sky-canopy border length, exposure and thresholding influence accuracy of hemispherical photography for complex plant canopies
Source: Bot Stud. 2018 Jul 28;59:19. doi: 10.1186/s40529-018-0235-9 (PMC6064577; doi:10.1186/s40529-018-0235-9)
Supplement: Supplementary file 1 — Additional file 1: Figure S1. Relationship between the sky-canopy border length and mixed-pixel zone length in model canopy hemispherical photographs. Figure S2. Using linear models to fit the relationships between the grey pixel fraction and canopy gap fraction in model canopy hemispherical photographs. Figure S3. Using linear and second order polynomial models to fit the relationships between the grey pixel fraction and canopy gap fraction in model canopy hemispherical photographs. Figure S4. Relationships of canopy gap fraction, the camera-to-canopy distance and lens’ view angle to the sky-canopy border length. Figure S5. Effects of the exposure-thresholding mismatch extents (ETM extents) on estimated gap fraction. Text S1. The method for obtaining the correct gap fraction for model canopies. Text S2. The impreciseness of our control on the light intensity of the light source when taking photographs for model canopies. [file 40529_2018_235_MOESM1_ESM.pdf]

Figure S1. Relationship between the sky-canopy border length and mixed-pixel zone length in model canopy hemispherical photographs. The unit for exposure manipulation is stop. Figure shows that pixel fraction of mixed-pixel zone is a close approximation of sky-canopy border length per unit area.

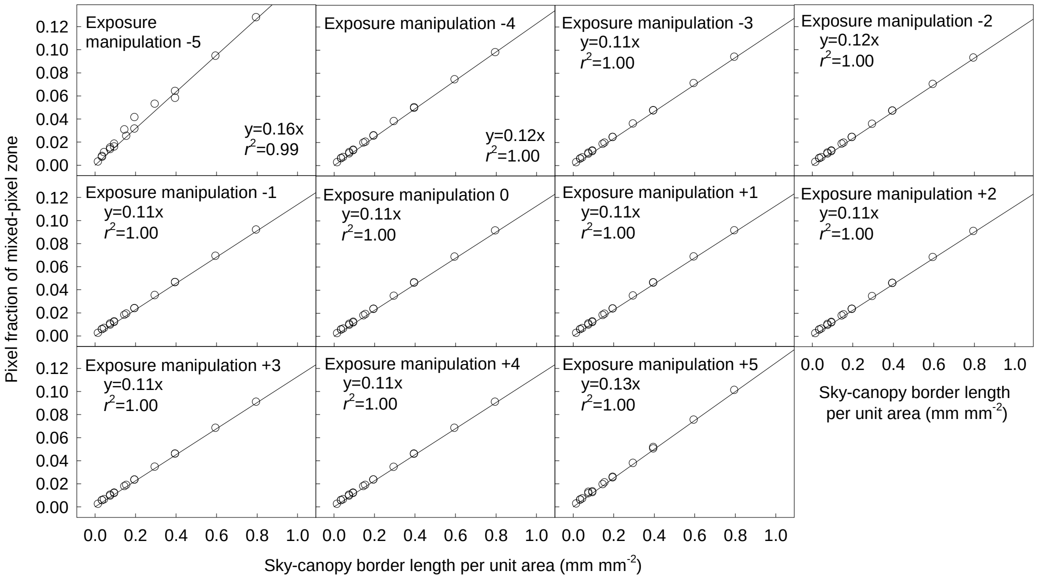

Figure S2. Using linear models to fit the relationships between the grey pixel fraction and canopy gap fraction in model canopy hemispherical photographs. The unit for exposure manipulation is stop. These relationships can be better fitted by hump-shaped models ($y= e^a x^b (1-x)^c$) in Fig. 2 than by linear models.

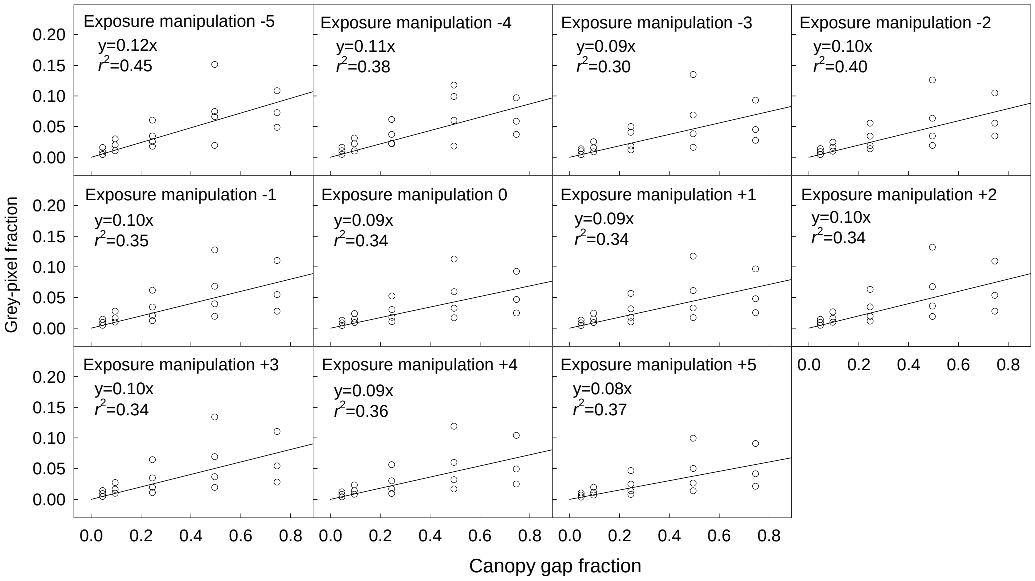

Figure S3. Using linear and second order polynomial models to fit the relationships between the grey pixel fraction and canopy gap fraction in model canopy hemispherical photographs. The unit for exposure manipulation is stop. These relationships can be better fitted by hump-shaped models ($y= e^a x^b (1-x)^c$) in Fig. 3 than by linear or second order polynomial models.

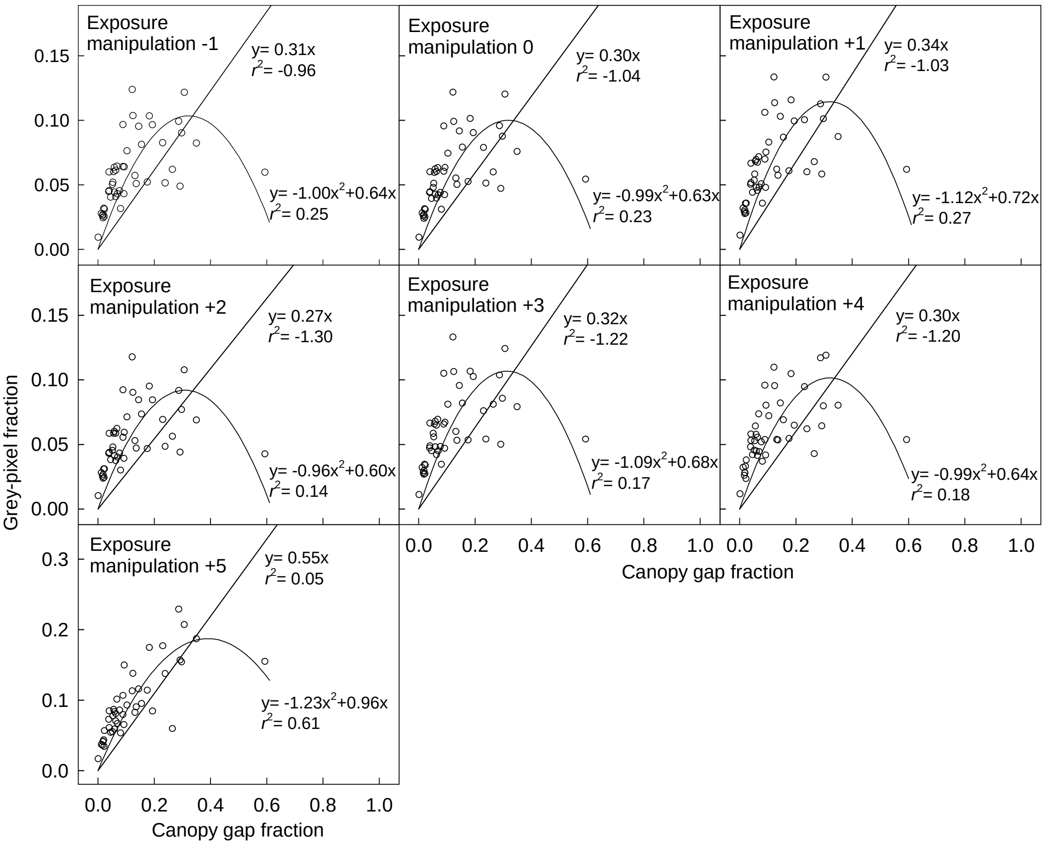

Figure S4. Relationships of canopy gap fraction, the camera-to-canopy distance and lens' view angle to the sky-canopy border length. This illustrative figure conceptually shows that 1) the sky-canopy border length in model canopies can be varied independently even their gap fraction are controlled, 2) doubling the camera-to-canopy distance can double the sky-canopy border length in acquired images, and 3) doubling the lens' view angle can also double the sky-canopy border length in acquired images.

Canopy gap fraction	Camera-to-canopy distance	Lens' view angle	Canopy images acquired	Sky-canopy border length in acquired images
0.25	1 unit	1n degree	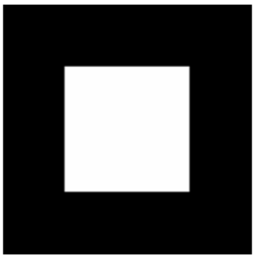	1 unit
0.25	2 unit	2n degree	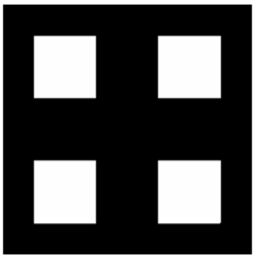	2 unit
0.25	4 unit	4n degree	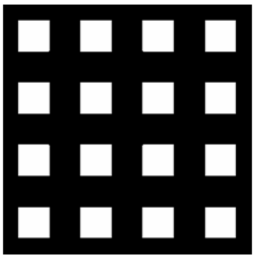	4 unit

Figure S5. Effects of the exposure-thresholding mismatch extents (ETM extents) on estimated gap fraction. These results are derived from real canopy hemispherical photographs (CHPs) taken in 46 locations. The exposure manipulation of these CHPs is +2 stop. Note log scale used on both x and y axes. The unit for the ETM extent is stop. If the ETM extent is > 0 stop, this means CHPs are overexposed or analysed with threshold values that are too low. If the ETM extent is < 0 stop, this means CHPs are underexposed or analysed with threshold values that are too high.

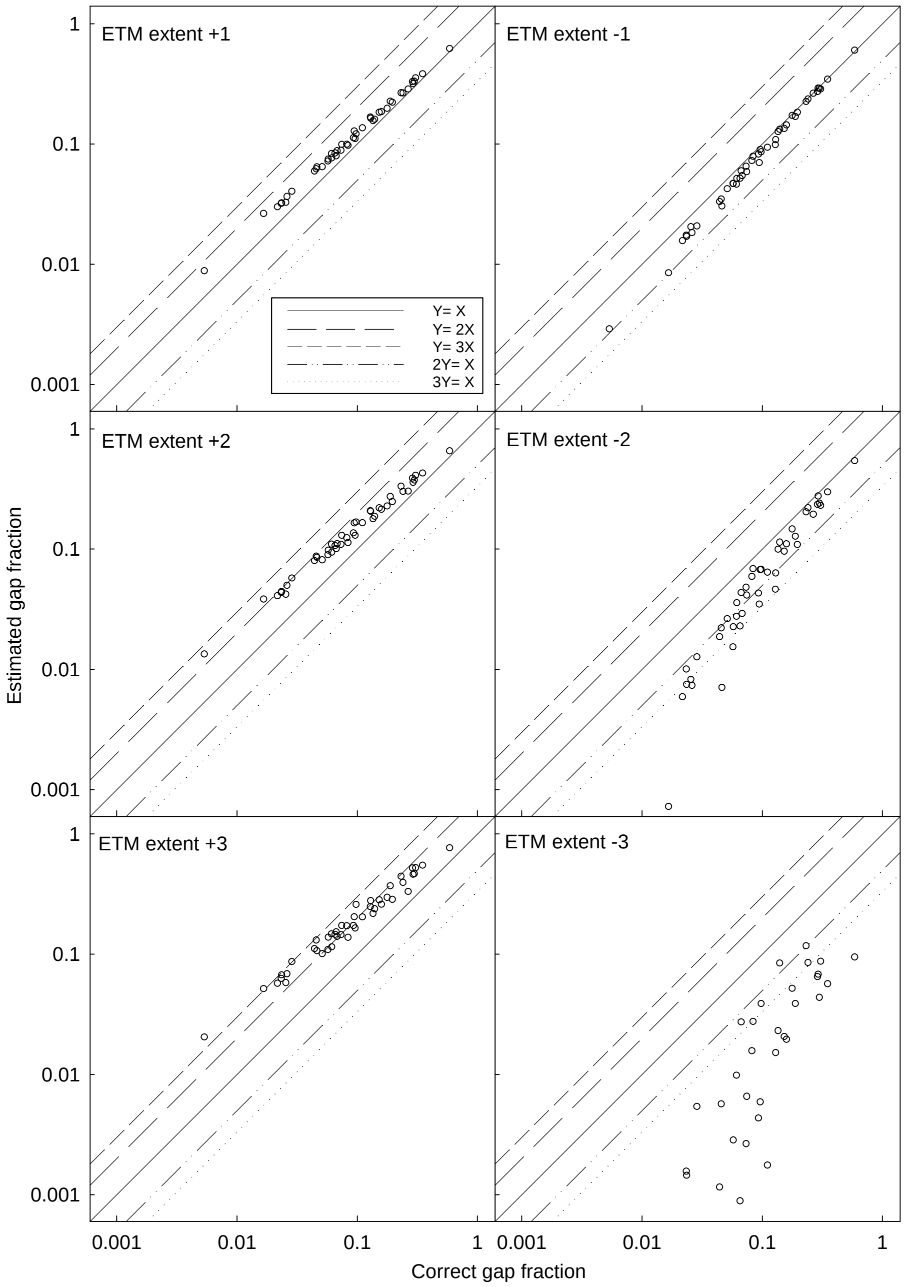

Text S1. The method for obtaining the correct gap fraction for model canopies.

An Epson Perfection V600 PHOTO scanner (Seiko Epson Corp., Suwa, Nagano, Japan) was used. Because the Epson V600 scanning program allows users to assign a threshold value and generate binary images straight away, we used the following method to identify the optimal threshold value. The model canopies with designed gap fraction of 0.25 and 2.5 mm side length of open squares were used due to its unique property. That is, the side length of open squares in this model canopy is equal to the width of black grids. The optimal threshold value is the one with which we can obtain an image with such property. Through comparing the side length of open squares and the grid width in scanned binary images with different threshold values, the optimal threshold value of 30 was identified. All of the 17 model canopies were scanned with threshold value of 30 and image resolution of 5714 x 5086 pixels and saved in the Jpeg image format. The amount of white and black pixels was acquired with the function of “Histogram” of ImageJ version 1.43 (Abramoff et al. 2004). The gap fraction of the scanned images was obtained through dividing the number of white pixels by that of all pixels.

Text S2. The impreciseness of our control on the light intensity of the light source when taking photographs for model canopies. Significant tests showed that HP estimation inaccuracy (*I*) vary significantly even though the ETM extent (*M*) was controlled (panels within the same rows in Fig. 9). This is attributed to that the control on the brightness of light source for the model canopy system was not precise enough. During the acquisition of model CHPs, the brightness of the light source could vary up to 0.3 stop due to the voltage variation of the alternating current power supply. The minimum scale of the light meter (Spotmeter F; Konica Minolta, Tokyo, Japan) with which we used to measure back illumination of the model canopy is 0.1 stop. If two model CHPs are taken with the light source brightness nearly 0.1 stop over and under what the light meter indicates respectively, the inconsistence of exposure manipulation between these two CHPs is nearly 0.2 stop. This inconsistence will be passed on to every parameter derived from exposure manipulation, including the ETM extent and slopes of regression lines between HP estimation inaccuracy and the ETM extent (Fig. 9). Apparently, even though the inconsistence of exposure manipulation is just nearly 0.2 stop, it is enough to result in significant differences among the slopes of those regression lines in Fig. 9. Future studies can use a light source with low power consumption (e.g. light-emitting diode) coupled with fully-charged large capacity batteries (e.g. batteries of electric cars) to reduce the brightness variation of light source.
